# Immunohistochemical Study of GATA3, c-KIT/CD117, CD56 and CD45 Expression in Proliferative Verrucous Leukoplakia (PVL), PVL-Associated Oral Squamous Cell Carcinoma and Oral Leukoplakia

**DOI:** 10.3390/genes16111275

**Published:** 2025-10-28

**Authors:** Eleni-Marina Kalogirou, Nikolaos Katsoulas, Dimitrios Goutas, Konstantina Vasili, Eleni Mikoglou, Theodora Tzanavari, Konstantinos I. Tosios

**Affiliations:** 1 Faculty of Health Sciences, Metropolitan College, 15125 Athens, Greece; kvasili22a@amcstudent.edu.gr (K.V.); emikoglou22b@amcstudent.edu.gr (E.M.); ttzanavari@mitropolitiko.edu.gr (T.T.); 2 Istomedica Anatomic Pathology Laboratory, 11528 Athens, Greece; nikolaoskatsoulas@gmail.com (N.K.); dimgoutas@med.uoa.gr (D.G.); 3 First Department of Pathology, School of Medicine, National and Kapodistrian University of Athens, 11527 Athens, Greece; 4 School of Dentistry, National and Kapodistrian University of Athens, 11527 Athens, Greece; ktosios@dent.uoa.gr

**Keywords:** proliferative verrucous leukoplakia, oral leukoplakia, oral potentially malignant disorder, squamous cell carcinoma, GATA3, c-KIT, CD117, CD56, CD45, immunohistochemistry

## Abstract

**Background/Objectives**: Proliferative verrucous leukoplakia (PVL) is an oral potentially malignant disorder characterized by a high risk for cancer development. Current evidence suggests that the evolution and malignant transformation of PVL is driven by a reciprocal crosstalk between the epithelial cells and the subepithelial immune microenvironment. The aim of the present study was to compare for the first time the immunohistochemical expression of the immune response-related proteins GATA-binding protein 3 (GATA3), c-KIT/cluster of differentiation (CD)117, CD56 and CD45 between PVL, PVL-associated oral squamous cell carcinoma (OSCC) and solitary (localized) oral leukoplakia (OL) cases. **Methods**: Thirty-six formalin-fixed and paraffin-embedded tissue specimens were used; sixteen from 8 patients with PVL, ten from 10 patients with PVL-OSCC and ten from 10 patients with OL. Immunohistochemistry was conducted using monoclonal primary antibodies against GATA3, c-KIT/CD117, CD56 and CD45. A semi-quantitative method was applied to score staining, and statistical analysis included Wilcoxon signed-rank test, Kruskal–Wallis test with Dunn’s post hoc test and Spearman’s correlation coefficient test. **Results**: A significantly decreased GATA3 expression was found in PVL-OSCC cases compared with PVL and OL cases. c-KIT/CD117 and CD56 proteins were consistently expressed in all study groups, while a significantly higher CD45 expression was noted in PVL than OL. No significant correlation between markers was found. **Conclusions**: These data collectively underscore an activated yet disturbed immune response that might be involved in the development and progression of malignancy in PVL that may also be considered as unique and interesting in vivo model of oral carcinogenesis.

## 1. Introduction

An oral potentially malignant disorder (OPMDs) is “any oral mucosal abnormality that is associated with a statistically increased risk of developing oral cancer” [1]. The overall prevalence of OPMDs worldwide is 4.47% [2]. OPMDs may involve any oral site, be focal or multifocal and present with alterations in the features of the normal oral mucosa, mostly in the color, e.g., white, red or any combination, and morphology, i.e., a plaque with smooth, corrugated, verrucous, granular, atrophic or ulcerated surface [1].

Oral leukoplakia (OL), the most common OPMD, is defined as “a predominantly white plaque of questionable risk (for developing cancer) having excluded (other) known diseases or disorders that carry no increased risk for cancer” [3]. The pathogenesis of OL involves environmental carcinogens, mostly tobacco smoking, followed by alcohol consumption and smokeless tobacco use, and in Asia the habit of chewing paan, khat or betel nut [4]. Most patients with OL are middle-aged or elderly males that smoke [1,5]. On clinical examination OLs present as white plaques, i.e., lesions that cannot be rubbed off, which may be homogeneous or non-homogeneous, i.e., speckled, nodular or verrucous. This classification has prognostic significance, as in a non-homogeneous OL there is a higher risk compared to homogeneous OL both for malignant transformation and for the biopsy to show severe dysplasia/carcinoma in situ or oral squamous cell carcinoma (OSCC) [1,6]. The pooled malignant transformation risk in OL is 3.5–9.8% (range 0.13% to 40.8%), the annual rate 1.56% [5] and the probability for malignant transformation is 6.64% (95% confidence interval  =  5.21–8.21) [7]. Non-homogeneous appearance, location on the lateral tongue or floor of the mouth, surface area > 200 mm^2^, female gender and older age (>50 years), as well as the presence of any grade of epithelial dysplasia are associated with a higher risk of malignant transformation [7,8].

Proliferative verrucous leukoplakia (PVL) is an OPMD [9] defined as a type, clinical form or subset of OL [8,10,11], or as a distinct spectrum disorder [12,13] characterized by a high propensity for malignant transformation. It was first documented in 1985 by Hansen et al. [14] who described it as a “continuum of hyperkeratosis ranging from a simple hyperkeratosis at one end to invasive SCC at the other” and suggested that similar cases had been earlier reported as “oral florid papillomatosis” [14]. The etiology of PVL is unclear, as known risk factors for OPMDs and OSCC, i.e., smoking and alcohol, are not strongly related to its development [9,15,16,17]. The typical patient with PVL is an elderly female that usually has none of the risk factors commonly associated with OL, such as smoking. Clinical examination shows adherent white lesions that may be multifocal (in topographically contiguous or adjacent sites) or multicentric (in topographically separate and distinct sites). The presentation may be heterogeneous: flat lesions consistent with homogeneous OL; fissured/verruciform, ulcerated or erythematous lesions consistent with non-homogeneous OL; exophytic, wart-like, verrucous lesions; and frank verrucous carcinoma and OSCC. The clinical course, as can be inferred by the patient’s recollection or documented by follow-up, is that of localized adherent white lesions that recur after (any kind of) intervention and extend over time, finally occupying a large area or multiple sites of the oral mucosa [10,12,16,17,18]. The gingiva/alveolar mucosa and buccal mucosa are the most commonly involved oral sites [13,18,19]. Malignant transformation in PVL was reported in systematic reviews to be from 45.8% [20] to 65.8% [21]. PVL-associated OSCC (PVL-OSCC) does not usually develop in the “classic areas” of lateral/ventral tongue, floor of mouth and soft palate, but prefers the gingiva and alveolar mucosa, buccal mucosa, hard palate and dorsal tongue [12,22,23,24], while in some studies gingival location has been associated with a high risk of malignant transformation [22,23].

Current evidence suggests that the evolution and malignant transformation of OPMDs is driven by a reciprocal crosstalk between the epithelial cells and the subepithelial microenvironment, which comprise nonmalignant cells, i.e., immune cells and fibroblasts, as well as extracellular matrix components, blood vessels and nerves, and undergoes dynamic remodeling during the disease progression [25,26,27]. Although immune cells, cytokines and cell-surface molecules are normally responsible for maintaining tissue homeostasis and immunosurveillance, the so-called “immune microenvironment” in OPMDs acquires a modified functional profile that favors immunosuppression and facilitates evasion of potentially malignant epithelial cells from host immune control [26,28]. Fundamental components of the immune microenvironment are innate immune cells, including macrophages, mast cells, dendritic cells, natural killer (NK) cells, neutrophils and myeloid-derived suppressor cells, as well as adaptive immune cells, such as cluster of differentiation (CD) 4^+^ T-helper (Th) cell subsets, i.e., Th1, Th2, Th17, T regulatory cells (T regs), CD8^+^ cytotoxic T cells and B cells [26,27,29,30]. One of the best-studied immune cell categories in OPMDs and OSCC are macrophages that switch from the M1 activation status that prevails in early stages of OPMDs to the M2 anti-inflammatory and pro-tumorigenic phenotype that is progressively adopted during the disease evolution [26,27,28,29,31]. M1 phenotype promotes the secretion of proinflammatory cytokines, e.g., interleukin (IL)-12, IL-23 and tumor necrosis factor-α (TNFα), and chemokines to attract adjacent immune cells, while M2 is triggered by Th2-released cytokines, e.g., IL-4, IL-10, IL-13 and predominates during malignant transformation [26,27,28,29,31].

T cells provide antigen-specific targeted responses and studies have shown controversial results, with both increased or decreased CD4+ and/or CD8+ T cell infiltration reported in OPMDs compared to OSCC [26,29]. Less studied immune cells in the setting of OPMDs and OSCC are mast cells and NK cells. Mast cells are considered to promote malignant transformation by inducing angiogenesis, while their density has been observed to increase from normal oral mucosa to OPMDs and OSCC [26,27,28,29]. Similarly, elevation in NK cell density has been reported during the transition from normal oral mucosa to OPMDs and OSCC [32]. NK cells have been found to exhibit multifaced anti-tumorous actions, e.g., by releasing cytotoxic molecules, including granzymes and perforin, promoting apoptosis and attracting neighboring immune cells, and, thus, represent promising targets of immunotherapy against OSCC [32].

The role of immune microenvironment in OL has been recently reviewed by González-Arriagada et al. [33] who highlighted the synergistic effect of M2 polarized macrophages, CD8+ T cells and T regs in promoting malignant transformation of OL. Moreover, studies utilizing immunohistochemistry alone [34] or in combination with cytokine and chemokine assays and flow cytometry [35], as well as studies based on advanced molecular techniques [36,37,38] have revealed a disrupted immune cell response in PVL, marked by increased expression of macrophages and cytotoxic T cells, as well as T lymphocyte subset imbalances, that favors immunosuppression. Purportedly, such abnormalities could be indicative of an increased potential for malignant transformation. Taken together, these studies underscore the role of the immune microenvironment in shaping the distinctly aggressive biological behavior of PVL among OPMDs. Further elucidating the composition of the immune cell subpopulations of PVL could advance our understanding of its pathogenesis and guide the design of targeted therapeutic strategies.

Thus, the aim of the present study was to compare for the first time the immunohistochemical expression of GATA-binding protein 3 (GATA3), a key regulator of Th2 cell differentiation [39], c-KIT/CD117, a marker of mast cells [40], CD56, which is predominantly expressed by NK cells [41], and the pan-leukocyte marker CD45 [42] between PVL, PVL-OSCC and OL cases.

## 2. Materials and Methods

### 2.1. Case Selection

Thirty-six formalin-fixed and paraffin-embedded (FFPE) tissue blocks were retrieved from the files of a private Pathology Laboratory. The three study groups consisted of (A) 16 blocks from 8 patients (2 blocks per patient) with PVL without malignant transformation; (B) 10 blocks from 10 patients with PVL-OSCC; and (C) 10 blocks from patients with solitary (localized) OL. All cases had been clinicopathologically diagnosed based on previously proposed criteria [1,21,43].

Group A (“PVL group”) included 16 FFPE samples derived from 7 females and 1 male, with age range from 49 to 73 years (mean age ± standard deviation (SD): 63.6 ± 8.1 years); one sample for each patient was from the gingiva and the other from the buccal mucosa (4/8) or tongue (4/8). Group B (“PVL-OSCC group”) consisted of 10 FFPE samples derived from 8 females and 2 males, with age range from 45 to 85 years (mean age ± SD: 70.7 ± 11.5 years), with PVL-associated OSCC involving the gingiva (5/10), the buccal mucosa (2/10), the hard palate (2/10) or the tongue (1/10). Group C (“OL group”) comprised 10 FFPE samples derived from 8 females and 2 males, with age range from 37 to 72 years (mean age ± SD: 58.9 ± 11.1 years), presenting with solitary leukoplakia that affected the tongue (3/10), the floor of mouth (3/10), the hard palate (2/10), the buccal mucosa (1/10) or the gingiva (1/10). Table S1 summarizes the patients’ demographic data and the location of lesions of the three study groups.

### 2.2. Immunohistochemistry

For immunohistochemical analysis, 3 μm thick sections were obtained from each FFPE tissue block using a Leica RM2145 microtome (Leica Biosystems Inc., Buffalo Grove, USA). Immunohistochemistry was carried out using the fully automated Leica BOND-MAX autostainer (Leica Biosystems, Melbourne, Australia), as previously described [44]. In brief, the biotin-free Bond Polymer Refine Detection System (DS9800, Leica Microsystems, Newcastle upon Tyne, UK) was used for antigen–antibody complex visualization, the 3,3-diaminobenzidine tetrahydrochloride solution as chromogen and Mayer’s hematoxylin as counterstain. The primary antibodies used were anti-GATA3 (mouse monoclonal, GenomeMe, IHC583, Richmond, BC, Canada; dilution 1:100; 30 min incubation in 95 °C), anti-CD117 (rabbit monoclonal, Cell Marque, YR145, Rocklin, CA, USA; 30 min incubation in 95 °C), anti-CD56 (rabbit monoclonal, GenomeMe, IHC056, Richmond, BC, Canada; dilution 1:100; 30 min incubation in 95 °C); and anti-CD45 (mouse monoclonal, GenomeMe, IHC045, Richmond, BC, Canada; dilution 1:100; 30 min incubation in 95 °C). The following tissue specimens were used as positive controls: urothelium for GATA3 [45], interstitial cells of Cajal for c-KIT/CD117 [46], ovarian stromal cells for CD56 [47] and lymphocytes in a lymph node section for CD45 [48]. For negative controls, the primary antibodies were substituted by non-immune serum of the same specificity.

### 2.3. Immunohistochemical Evaluation

Immunohistochemical staining across the entire tissue section was assessed by two observers (E.-M.K., K.I.T.) independently, and cases with discrepancies in scoring were resolved through consensus discussion. Nuclear staining for GATA3 [49] and membranous/cytoplasmic staining for c-KIT/CD117 [50], CD56 [51] and CD45 [52] were regarded as positive. For scoring, a semi-quantitative method was applied that incorporated the intensity of staining and the percentage of positive cells that were classified according to previously proposed scoring systems for each protein marker [49,50,51,52]. A final score was obtained as the product of the two individual scores for extent and intensity of staining (Table 1). In PVL-OSCC group the expression of all markers was scored in the malignant epithelial cells. The stained tissue section of each slide was scanned using the digital scanner Pannoramic MIDI II (3DHISTECH; Budapest, Hungary) and representative images from each section were captured.

### 2.4. Statistical Analysis

Descriptive statistical analysis was performed to evaluate the demographics and the site of lesions in all study groups. The final score of all study cases was assessed for normality using the Shapiro–Wilk test. Due to the ordinal nature of data, i.e., final score, and the small number of cases per group, non-parametric analysis techniques were applied. As PVL group included 2 samples per patient, the final score of immunohistochemical expression of each marker was calculated for each one of the two samples and the Wilcoxon signed-rank test for matched pairs was used to compare the final score between paired samples (gingiva vs. other site). Because ties and zero differences were present, exact *p*-values were not available and asymptotic *p*-values without continuity correction were reported. Holm correction was applied to adjust for multiple testing across the four markers and effect sizes were measured using the rank-biserial correlation (r). Next, the mean score across both samples of each patient of PVL group was computed, as previously recommended [53,54] and was rounded to zero decimal places, to reflect the ordinal scale of scoring. The Kruskal–Wallis test with Dunn’s multiple comparison post hoc test (two-sided, tie-corrected z) with Bonferroni adjustment was performed to compare the final score between the three study groups. Spearman’s correlation coefficient test with Bonferroni correction to adjust for multiple comparisons was used to assess the correlation between the final score of the four markers in each study group and in the total samples. Statistical analysis was computed using R Studio (R version 4.3.1). *p*-value < 0.05 was set as statistically significant.

## 3. Results

**Figure 1:**
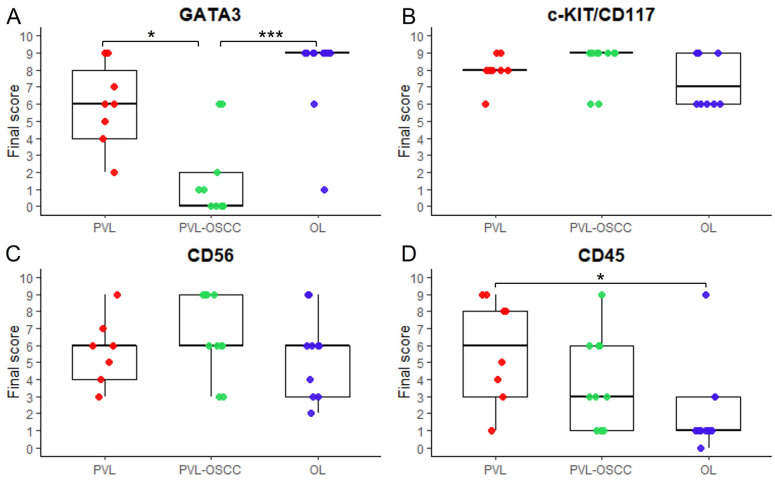
Final score of (**A**) GATA3, (**B**) c-KIT/CD117, (**C**) CD56 and (**D**) CD45 in PVL, PVL-OSCC and OL groups. Boxplots illustrate the medians and interquartile range (Q1–Q3) based on Tukey hinges (rounded down to the closest integers), while whiskers extend to the most extreme values within 1.5× interquartile range (rounded to the closest integers). Each dot corresponds to the final score of each patient in PVL (red dots), PVL-OSCC (green dots) and OL (blue dots) groups. *p*-values after Bonferroni adjustment < 0.05 and <0.001 are indicated by * and ***, respectively.

The final score of all markers in each study case is depicted in Figure 1. Intensity and extent score in each study case are provided in detail in Table S2, while the final score of each marker in all study groups is presented as median and interquartile (Q1–Q3) range using Tukey’s hinges and rounded down to the closest integers, in line with ordinal nature of data, in Table S3. The Wilcoxon signed-rank tests with Holm correction revealed no statistically significant differences in the final score of all tested markers between the two paired samples of each patient in PVL group (Table S4). Moreover, Spearman’s correlation coefficient tests with Bonferroni adjustment for multiple comparisons resulted in no significant correlation between the final score of the four markers in any study group, as well as in the total study cases (Table S5).

### 3.1. GATA3 Immunohistochemical Expression

**Figure 2:**
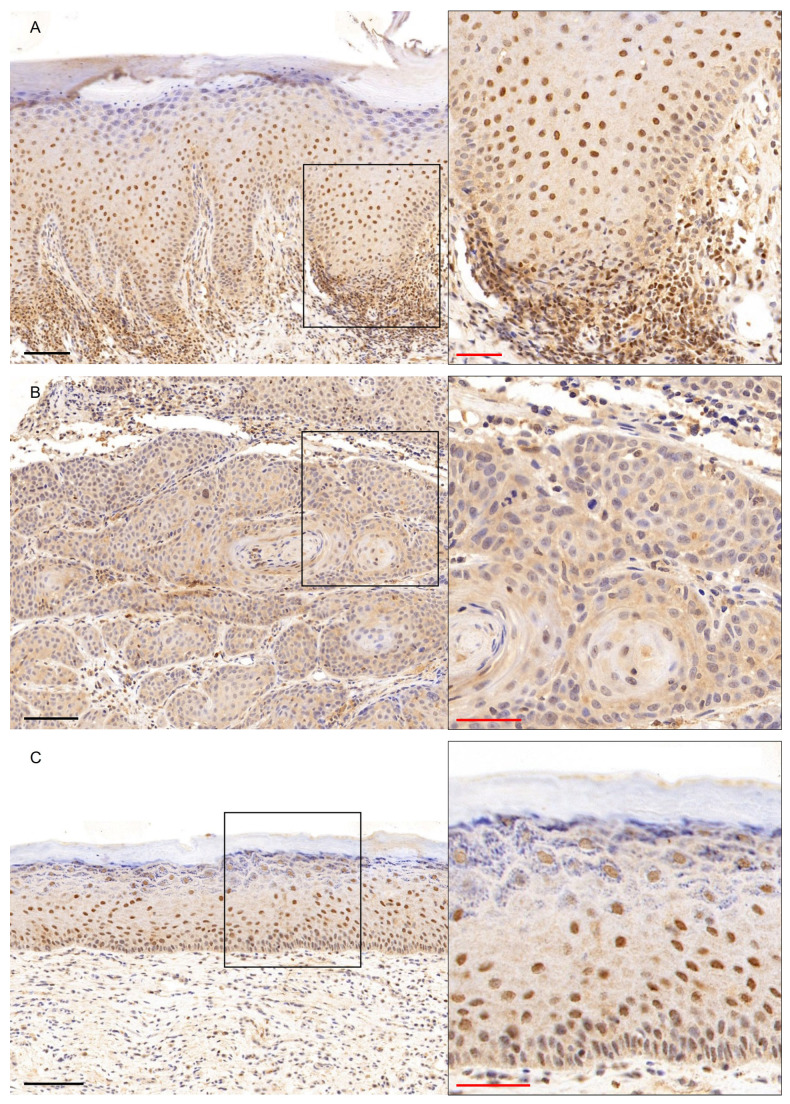
GATA3 immunohistochemical expression in (**A**) PVL, (**B**) PVL-OSCC and (**C**) OL groups. Black scale bars = 100 μm, red inset scale bars = 50 μm. Black rectangle corresponds to the magnified area.

GATA3 showed moderate to strong nuclear expression in the epithelial cells of basal and predominantly suprabasal layers in all cases of PVL (Figure 2A), with 7/8 PVL cases presenting a final score of ≥4 (Figure 1A, Table S2), while nuclear staining was also observed in inflammatory cells in the subepithelial connective tissue (Figure 2A). In contrast, 5/10 and 3/10 cases of PVL-OSCC group were negative or mild positive, respectively (Figure 1A, Table S2), with malignant cells showing no staining or only focal nuclear positivity (Figure 2B). Strong GATA3 immunostaining was found in the epithelial cells of basal and mainly suprabasal layers, while moderate nuclear staining was seen also in cells of the granular epithelia layer (Figure 2C) in OL group, with a final score ≥ 6 in 9/10 OL cases (Figure 1A, Table S2). The Kruskal–Wallis test revealed a statistically significant difference in the expression of GATA3 between the three groups (*p*-value = 0.000326, Table S3). The Dunn’s multiple comparison post hoc test with Bonferroni adjustment of *p*-values showed a significantly lower GATA3 expression in PVL-OSCC group compared with PVL (*p*-adjusted = 0.0493) or with OL group (*p*-adjusted < 0.001), while no significant difference was observed between PVL and OL groups (*p*-adjusted = 0.5442, Table S3).

### 3.2. c-KIT/CD117 Immunohistochemical Expression

**Figure 3:**
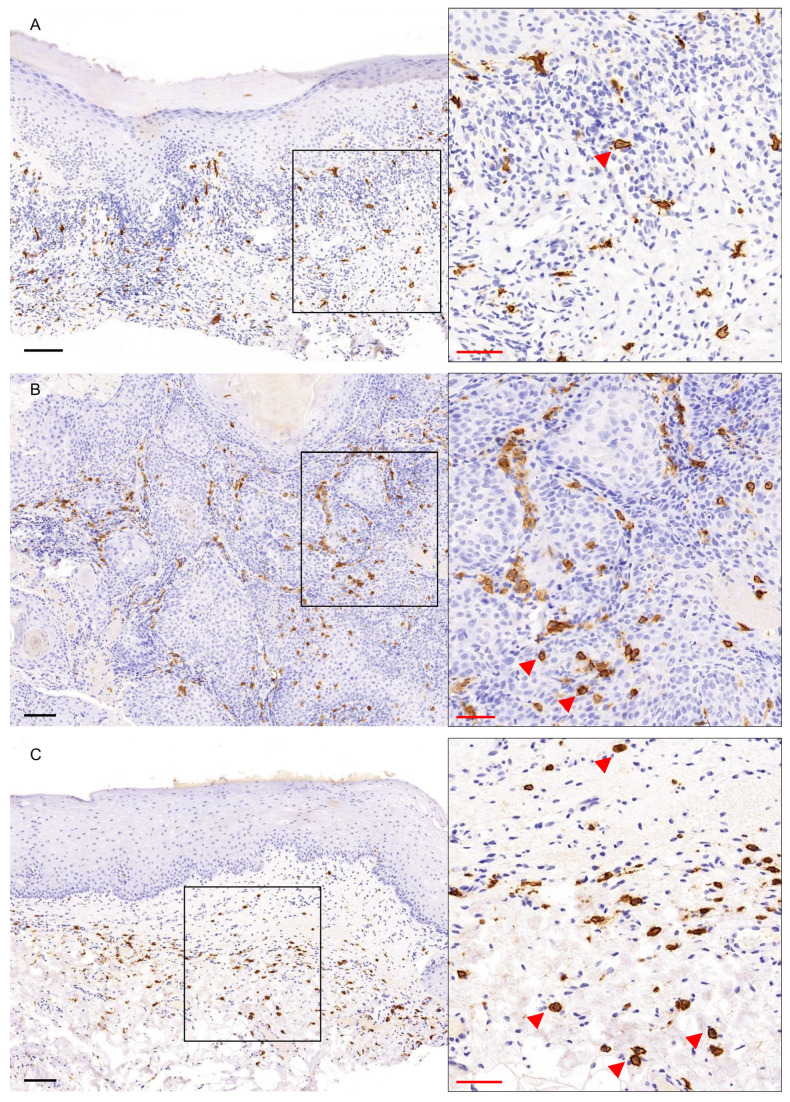
c-KIT/CD117 immunohistochemical expression in (**A**) PVL, (**B**) PVL-OSCC and (**C**) OL groups. Black scale bars = 100 μm, red inset scale bars = 50 μm. Black rectangle corresponds to the magnified area.

c-KIT/CD117 showed strong membranous/cytoplasmic expression in all study groups and a final score ≥6 was found in all cases (Figure 1B, Table S2). The immunoexpression of c-KIT/CD117 was noted in cells of the papillary and reticular lamina propria in PVL (Figure 3A) and OL (Figure 3C) cases, as well as in the connective tissue cells between the neoplastic epithelial islands in PVL-OSCC group (Figure 3B). Immunostaining was seen in cells of different shapes, i.e., spindle, polygonal or, as well as in oval cells with centrally located round to oval nuclei, consistent with mast cells [55], (Figure 3, red arrows). In addition, focal c-KIT/CD117 positivity was observed in the basal epithelial cells in some PVL and OL cases (Figure S1A), as well as in few intraepithelial cells in PVL-OSCC cases (Figure S1B, black arrows). According to Kruskal–Wallis test, c-KIT/CD117 expression did not differ significantly among PVL, PVL-OSCC and OL groups (*p*-value = 0.237, Table S3).

### 3.3. CD56 Immunohistochemical Expression

**Figure 4:**
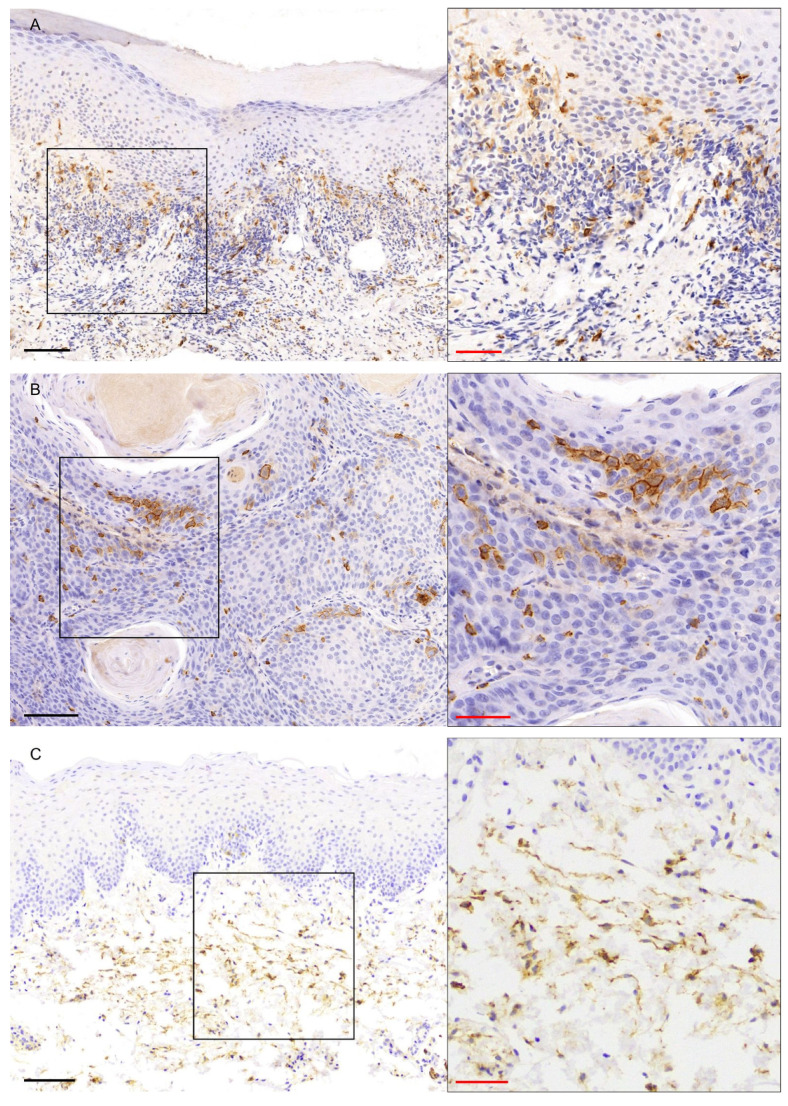
CD56 immunohistochemical expression in (**A**) PVL, (**B**) PVL-OSCC and (**C**) OL groups. Black scale bars = 100 μm, red inset scale bars = 50 μm. Black rectangle corresponds to the magnified area.

A moderate to strong membranous/cytoplasmic CD56 expression was found in all study cases (Figure 1C, Table S2). In PVL (Figure 4A) and OL (Figure 4C) cases, CD56 immunostaining was predominantly observed in cells in the subepithelial connective tissue, with scarce positive intraepithelial cells, with 7/8 cases and 7/10 cases presenting a final score ≥ 4 in PVL and OL group, respectively (Figure 2C, Table S2). In PVL-OSCC group, CD56 expression was seen in the malignant epithelial cells forming neoplastic chords and islands, as well as in cells in the connective tissue between the neoplastic epithelial islands (Figure 4B), with 8/10 PVL-OSCC cases presenting a final score ≥ 6 (Figure 2C, Table S2). No significant difference in the final score of CD56 was found between PVL, PVL-OSCC and OL groups by Kruskal–Wallis test (*p*-value = 0.486, Table S3).

### 3.4. CD45 Immunohistochemical Expression

**Figure 5:**
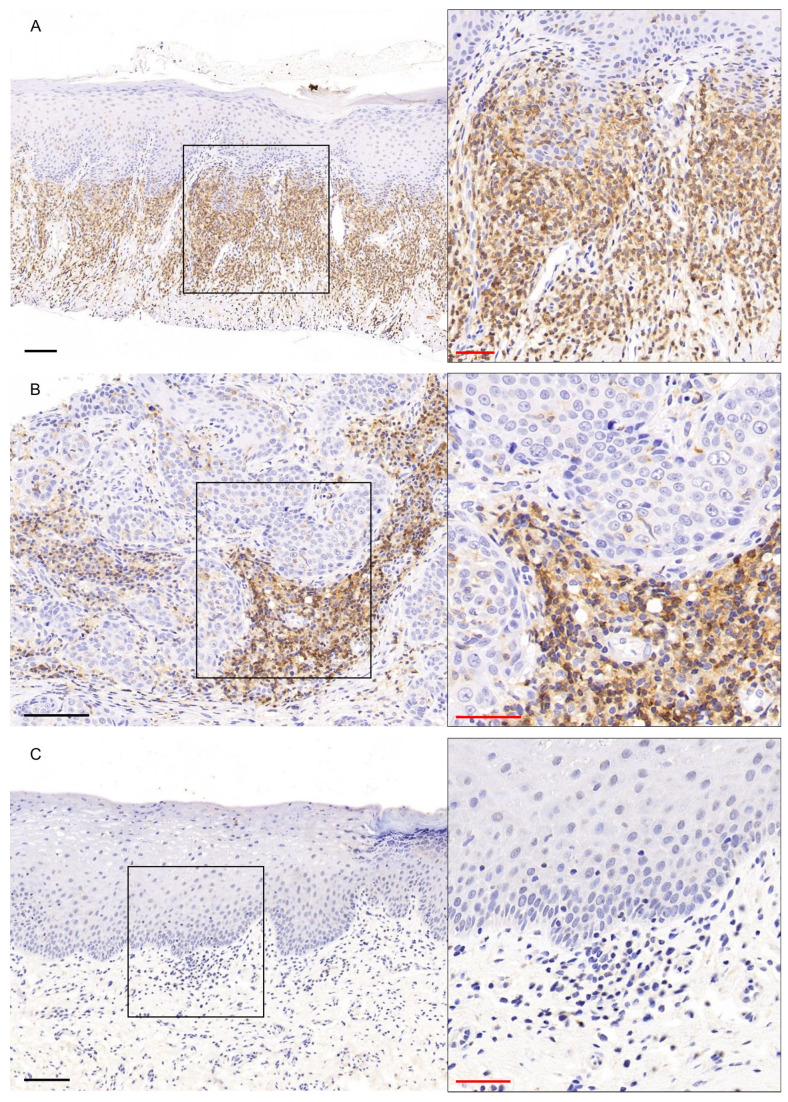
CD45 immunohistochemical expression in (**A**) PVL, (**B**) PVL-OSCC and (**C**) OL groups. Black scale bars = 100 μm, red inset scale bars = 50 μm. Black rectangle corresponds to the magnified area.

CD45 showed strong membranous/cytoplasmic expression in dense, band-like distribution in subepithelial location in PVL group (Figure 5A), with 6/8 PVL cases presenting a final score ≥ 4 in PVL (Figure 1D, Table S2). In PVL-OSCC cases, moderate to strong membranous/cytoplasmic staining of CD45 was observed in focal areas of connective tissue cells between the neoplastic epithelial islands, while mild expression was observed in few intraepithelial cells (Figure 5B), with 6/10 PVL-OSCC cases presenting a final score of 1 to 3 (Figure 2, Table S2). Mild membranous/cytoplasmic CD45 staining in few connective tissue cells was found in OL group (Figure 5C), with 7/10 OL cases presenting a final score of 1 to 3, while 1 OL case was negative for CD45 (Figure 2, Table S2). The Kruskal–Wallis test resulted in a statistically significant difference in the expression of CD45 between the three groups (*p*-value = 0.0247, Table S3). The Dunn’s multiple comparison post hoc test with Bonferroni adjustment of *p*-values revealed a significantly higher CD45 expression in PVL group compared with OL group (*p*-adjusted = 0.0211) while no significant differences were found between PVL and PVL-OSCC groups (*p*-adjusted = 0.7036, Table S3), as well as between OL and PVL-OSCC groups (*p*-adjusted = 0.3307, Table S3).

## 4. Discussion

**Figure 6:**
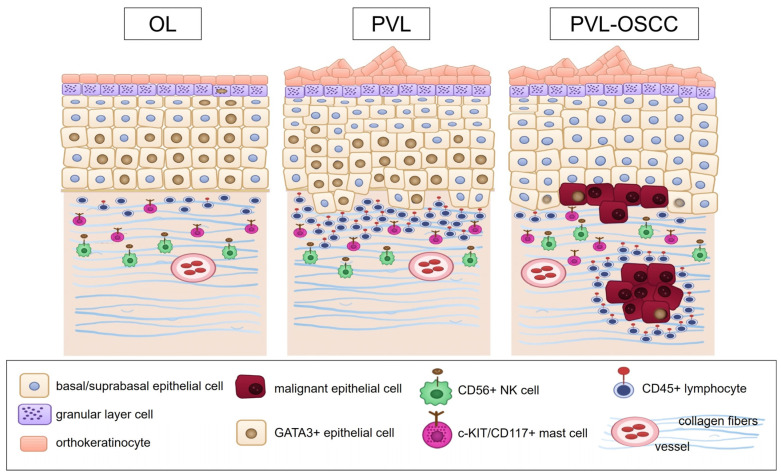
This schematic diagram illustrates the expression of GATA3, c-KIT/CD117, CD56 and CD45 in PVL, PVL-OSCC and OL. In PVL and OL, strong GATA3 expression was observed in the epithelial cells, as well as the subepithelial compartment, probably reflecting an activated Th2 response [56,58,59,60], whereas markedly decreased GATA3 expression was noted in PVL-OSCC, suggesting loss of immune control. c-KIT/CD117 was consistently expressed across all study groups, indicative of mast cells’ presence and their possible contribution to microenvironment modulation and activation of oncogenic signaling pathways [74,75]. CD56 was predominantly expressed in stromal cells in all cases, probably highlighting the activity of NK cells in enhancing the adaptive immunity and promoting anti-tumor effects [41]. Finally, a significantly higher CD45 expression was noted in PVL than OL, suggestive of intense inflammatory infiltration that might be related to the more aggressive behavior of PVL [35]. Taken together, these findings are indicative of an activated yet disturbed immune response that might be involved in the increased risk for development and progression of malignancy in PVL. The image was created with Inkscape v1.4.2, retrieved from https://inkscape.org (accessed on 26 September 2025).

Herein, we report for the first time comparative immunohistochemical data regarding the expression of GATA3, c-KIT/CD117, CD56 and CD45 among patients with PVL, PVL-OSCC and OL. Figure 6 illustrates a schematic diagram summarizing the key expression findings and depicting a proposed role of the evaluated markers.

An intense GATA3 epithelial expression was observed in PVL and OL cases. GATA3 is a zinc-finger transcription factor involved in diverse cellular processes that promote proliferation and differentiation [56,57]. GATA3 expression has been identified in various developing and normal adult tissues, including the epidermis, oral epithelium, sebaceous epithelia, hair follicles of the skin, luminal epithelium of mammary glands, parathyroid glands, kidneys and urothelium [56,58,59,60]. In normal epidermis, more intense expression of GATA3 is noted in the cells of the superficial layers, compared to the basal layer [60]. The predominant expression of GATA3 by differentiated squamous epithelial cells of the suprabasal epithelial layers in PVL and OL cases in the present study is consistent with those findings. GATA3 is also expressed by Th2 lymphocytes and exerts a critical role in early T cell commitment, CD4+ T cell development and Th2 differentiation [56,58,59,60]. An esteemed research group from Spain, utilizing RNA-sequencing [37] and methylated DNA immunoprecipitation coupled with high-throughput sequencing [61] on oral biopsies from PVL patients and healthy donors, identified GATA3 among the significantly upregulated and differentially methylated genes, respectively, in PVL, and suggested a possible role of GATA3 in the cancer-promoting immune response in PVL. The strong GATA3 expression in the inflammatory cells in the subepithelial connective tissue of PVL cases found in the present study might be indicative of the contribution of GATA3 to the immune microenvironment of PVL.

Most PVL-OSCC cases in the current study were negative or showed a focally mild positivity for GATA3. This finding agrees with a previous microarray-based study evaluating GATA3 expression in 127 OSCCs that found negative staining in 93.7% of them [60], as well as with an immunohistochemical study reporting GATA3 positivity only in 5 out of 15 head and neck SCCs (HNSCCs) [62]. Another immunohistochemical study utilizing GATA3 monoclonal antibody (1:100, Santa Cruz), reported high GATA3 expression in only 31% of 151 HNSCCs, including 29 out of 102 OSCCs with high GATA3 expression [63]. In this study, high GATA3 expression was significantly correlated with adverse prognostic factors, i.e., lymph node and distant metastasis, high grade, perineural invasion, lymphovascular invasion, extracapsular spread and presence of locoregional recurrence, and was an independent predictor of poor disease-free survival [63]. This was attributed to expression of genes involved in cancer progression and invasiveness through stabilization of hypoxia-inducible factor 1-alpha under hypoxic conditions that promotes tumor invasiveness [63]. Interestingly, PVL-OSCC shows considerably lower mortality than non PVL-OSCC [17,64,65]; in one meta-analysis it was found to be 21.3% vs. 34.7–50% [65]. Furthermore, gingival PVL-OSCC are of low stage (usually Stage I) and this has been attributed to early detection due to regular follow-up [22]. The low expression of GATA3 in PVL-OSCC found in the present study is another factor that may be associated with the better biological behavior of those lesions. Decreased GATA3 immunoexpression has also been reported in SCC of other origin, including those of pharynx, larynx, lung and cervix [59,60]. In addition, previous studies documented a significantly lower GATA3 expression in cancer, for example, of the skin [66], cervix [67] and vagina [68], compared with their respective premalignant lesions. These observations strongly align with our findings of a significantly decreased GATA3 expression in epithelial cells of PVL-OSCC cases compared to PVL and OL cases. They collectively point to the potential role of GATA3 in malignant transformation of OPMDs, mostly PVL, and warrant further investigation.

We also found significantly higher CD45 expression in PVL cases compared to OL samples. High CD45 expression score was also noted in the PVL-OSCC group, but statistical analysis failed to detect a significant difference with OL group, probably due to the sample size. CD45, also known as leukocyte common antigen, is a protein receptor tyrosine phosphatase type, expressed in the surface of almost all hematopoietic cells, with a crucial role in regulating the antigen receptor-mediated activation of lymphocytes [69]. Previous studies highlighted CD45 as an indirect marker of tumor-infiltrating lymphocytes (TILs). A significant positive correlation between CD45 expression, measured by real-time polymerase chain reaction, and TILs score, counted on hematoxylin–eosin stain, in HNSCCs has been reported [70]. In the later study [70], high CD45 expression was identified as a favorable prognostic factor associated with better local recurrence-free survival and disease-specific survival in patients with HNSCCs. Similarly, an immunohistochemical study found on univariate, but not in multivariate analysis, that high density of CD45-positive TILs was associated with better overall survival of OSCC [71]. In the same study [71], a significantly higher CD45 expression was observed among OSCC with increased score of the programmed death-ligand 1 (PD-L1), an immune checkpoint protein that promotes immunosuppression via binding to the PD-1 receptor on T cells [72]. A remarkable study by Hanna et al. [36], utilizing NanoString Immune Gene Expression Analysis to compare the immunoprofile of PVL and OL, found a significantly higher expression of CD8+ T cells, T regs as well as PD-L1 in PVL and proposed the immunotherapy targeting the PD-1/PD-L1 pathway might be of therapeutic benefit in PVL. The same research group evaluated the anti-PD-1 immune checkpoint drug nivolumab (480 mg every 4 weeks) to 33 patients with high-risk PVL [73]. Among the patients that completed the trial 36% showed clinical-pathologic regression, the cancer-free survival rate was 2 years for approximately 70% of the patients, but 27% developed OSCC [73]. The well documented activation of immune response in PVL [34,35,36,37,38], and the findings of the present study, suggest that additional research is required to elucidate the potential effect of immunotherapy.

Strong immunoexpression of c-KIT/CD117 in stromal cells with morphological characteristics consistent with mast cells, was a common finding in our three study groups. c-KIT/CD117 is a transmembrane protein encoded by the KIT Proto-Oncogene that belongs to the family of receptor tyrosine kinases and participates in important biological processes, including cell proliferation, apoptosis, differentiation, cell adhesion and chemotaxis [74]. It is expressed in a variety of normal cells, such as mast cells, hematopoietic stem cells and Cajal cells of the gastrointestinal tract, as well as in neoplastic tissues, associated with the activation of known oncogenic signaling pathways, e.g., the phophatidylinositol-3-kinase/Akt pathway and the Ras/Mitogen-Activated Protein kinase pathway [74,75]. c-KIT/CD117 has been employed as a marker of mast cells in previous studies evaluating its immunoexpression in OL and/or OSCC, occasionally with contradicting results. Oliveira-Neto et al. [76] found decreased expression of c-KIT/CD117 positive mast cells in OL and OSCC compared with normal oral mucosa, whereas two other studies, using different anti-CD117 antibodies, reported the opposite finding [77,78]. Low mast cell density, identified via decreased c-KIT/CD117 immunopositivity, has been reported as an adverse prognostic factor in OSCC and HNSCC, correlating with advanced histopathological grade [79], recurrence [80] and poor overall survival [81]. Moreover, c-KIT/CD117 expression has been previously observed in basal epithelial cells of OL [78,82], as well as in neoplastic epithelial cells of HΝSCC [83]. These findings are also confirmed by our results. To the best of our knowledge, the immunoexpression of c-KIT/CD117 in PVL cases has not been documented before. As mast cells participate in tumor immune response in OSCC [84] and have identified among immune cell populations in PVL by single cell RNA-sequencing [38], their role in PVL merits further investigation.

In the present study, all cases examined expressed CD56 predominantly in stromal cells, but also sporadically in epithelial cells of PVL and OL, and neoplastic epithelial cells in some PVL-OSCC cases. CD56, also known as neural cell adhesion molecule, is a membrane-bound cell-surface molecule that participates in various biological processes, including cell adhesion, migration and intercellular signaling [41,85]. It is predominantly expressed by NK cells, as well as in subsets of T cells, dendritic cells and few monocytes, and is involved in the induction of both innate and adaptive immune responses [41]. NK cells exert anti-tumor effects via several mechanisms, including promotion of apoptosis and secreting cytokines and chemokines that further amplify the adaptive immunity [86], and CD56 is essential to the retention of their migration properties [85]. CD56 expression has been previously shown in OSCC [87], oropharyngeal SCC [88] and HNSCC [89], where a high CD56 immunopositivity was shown to be a favorable prognostic factor. As PVL-OSCCs show a less aggressive biological profile compared to non PVL-OSCCs [17,64,65], CD56 expression that was revealed in PVL-OSCCs in our study may reflect this favorable clinical phenotype. To the best of our knowledge, this is the first study to assess CD56 immunostaining in PVL. As recent research supports the promising effects of NK cell-based immunotherapies against OSCC [32], the need for further studies to elucidate the expression and role of CD56 in PVL seems warranted.

No significant correlation was found between the four immune-related markers evaluated in the present study, but the moderate to strong expression of all of them in most PVL cases is in agreement with the concept of an enriched, activated, though probably dysregulated immune environment in PVL that forms a premalignant niche prone to malignant transformation, as has been previously suggested [37]. Gingival location of PVL has been associated with the highest risk for malignant transformation [22,23]. However, in this study no significant difference was found in the expression of any of the four immunohistochemical markers evaluated between gingival PVL and non-gingival PVL lesions. Investigation of those and additional markers in larger patient cohorts is needed to elucidate the phenotype of gingival PVL that may be associated with its increased malignant propensity.

A strength of our study is the comparative immunohistochemical evaluation of GATA3, c-KIT/CD117, CD56 and CD45 among PVL, PVL-OSCC and OL that was performed for the first time. The loss of GATA3 expression in PVL-OSCC cases highlighted in the present study may be considered a clinically significant finding, as, if confirmed in larger cohorts, this could establish GATA3 as a negative differentiation marker, with prospective applications in the differential diagnosis of oral epithelial dysplasia and carcinoma. Although the small sample size may be considered as a limitation of this study, it was sufficient to allow for statistical significance to be reached. An additional limitation is the cross-sectional study design that did not allow the expression of the four markers to be correlated with the risk for malignant transformation in the two groups of OPMDs. Future research should include a larger patient cohort and employ multi-plex immunostaining methods coupled with advanced molecular techniques, to unravel in-depth the immune microenvironment imprint on PVL and its associated malignancy. Inclusion of patients with available outcomes on recurrence or malignant transformation would allow us to draw robust conclusions regarding the potential impact of immune-related molecules on the biological behavior of OPMDs.

In conclusion, this study assessed the immunoexpression of GATA3, c-KIT/CD117, CD56 and CD45 among patients with PVL, PVL-OSCC and OL. A significantly decreased GATA3 expression was found in PVL-OSCC cases compared with PVL and OL cases. c-KIT/CD117 and CD56 proteins were consistently expressed in all study groups, while a significantly higher CD45 expression was noted in PVL than OL. These data collectively underscore an activated yet disturbed immune response that might be involved in the development and progression of malignancy in PVL that may also be considered as unique and interesting in vivo model of oral carcinogenesis.

## Figures and Tables

**Table 1:** Immunohistochemical scoring systems for each protein marker.

Protein Marker	Applied Scoring System	Extent Score ^1^	Intensity Score
GATA3	Liang et al. 2014 [49]	0 (0%), 1 (1–10%), 2 (11–50%), 3 (51–80%), 4 (>80%)	0 (negative), 1 (weak), 2 (moderate), 3 (strong)
c-KIT/CD117	Goto et al. 2016 [50]	0 (<1%), 1 (1–4%), 2 (5–29%), 3 (≥30%)	0 (negative), 1 (weak), 2 (moderate), 3 (strong)
CD56	Pallavi et al. 2025 [51]	0 (0%), 1 (1–9%), 2 (10–20%), 3 (21–50%), 4 (>50%)	0 (negative), 1 (weak), 2 (moderate), 3 (strong)
CD45	Pantanowitz et al. 2025 [52]	0 (0%), 1 (1–24%), 2 (25–49%), 3 (50–74%), 4 (75–99%), 5 (100%)	0 (negative), 1 (weak), 2 (moderate), 3 (strong)

^1^ Parentheses correspond to percentages of positive cells.

## Data Availability

The original contributions of the study are included in the article and Supplementary Materials. Further inquiries can be directed to the corresponding author.

## Supplementary Materials

The following supporting information can be downloaded at https://www.mdpi.com/article/10.3390/genes16111275/s1. Figure S1: c-KIT/CD117 expression in the basal epithelial cells in (A) OL and (B) PVL-OSCC cases; Table S1: Patients’ demographics and lesions’ site of all study cases; Table S2: Intensity score, extent of staining score and final score for all study cases, as well as mean final score for each pair of samples of PVL group; Table S3: Comparison of GATA3, c-KIT/CD117, CD56 and CD45 final scores [median (Q1–Q3), mean of ranks] between PVL, PVL-OSCC and OL groups using the Kruskal–Wallis test with Dunn’s post hoc comparisons and Bonferroni adjustment; Table S4: Comparison of GATA3, CD117, CD56 and CD45 expression between gingival PVL and PVL in other sites using Wilcoxon signed-rank test with Holm correction; Table S5: Spearman’s Correlation Coefficients (ρ) with Bonferroni adjustment for marker pairs across the total cases and each study group.

## Abbreviations

CD, Cluster of Differentiation; FFPE, Formalin-Fixed Paraffin-Embedded; GATA3, GATA-Binding Protein 3; HNSCC, Head and Neck Squamous Cell Carcinoma; IL, Interleukin; NK, Natural Killer; OL, Oral Leukoplakia; OPMD, Oral Potentially Malignant Disorder; OSCC Oral Squamous Cell Carcinoma; PD-L1, Programmed Death-Ligand 1; PVL, Proliferative Verrucous Leukoplakia; PVL-OSCC, Proliferative Verrucous Leukoplakia-associated Oral Squamous Cell Carcinoma; Q, Quartile; SCC, Squamous Cell Carcinoma; SD, Standard Deviation; T-reg, T- regulatory cell; TIL, Tumor-Infiltrating Lymphocyte; TNF-α, Tumor Necrosis Factor-alpha; Th, T-helper.
