# Supplementary figures and images for: Immunohistochemical Study of GATA3, c-KIT/CD117, CD56 and CD45 Expression in Proliferative Verrucous Leukoplakia (PVL), PVL-Associated Oral Squamous Cell Carcinoma and Oral Leukoplakia
Source: Genes (Basel). 2025 Oct 28;16(11):1275. doi: 10.3390/genes16111275 (PMC12652745; doi:10.3390/genes16111275)

A

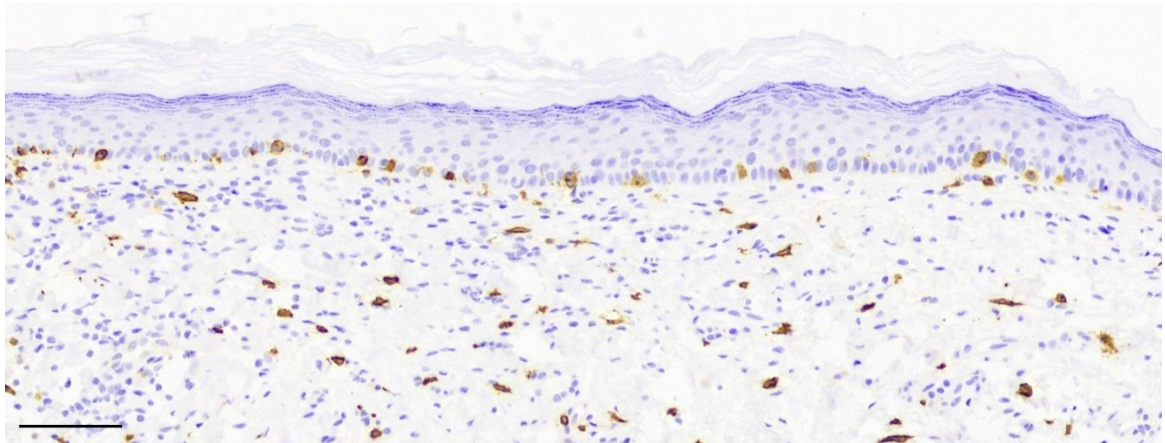

B

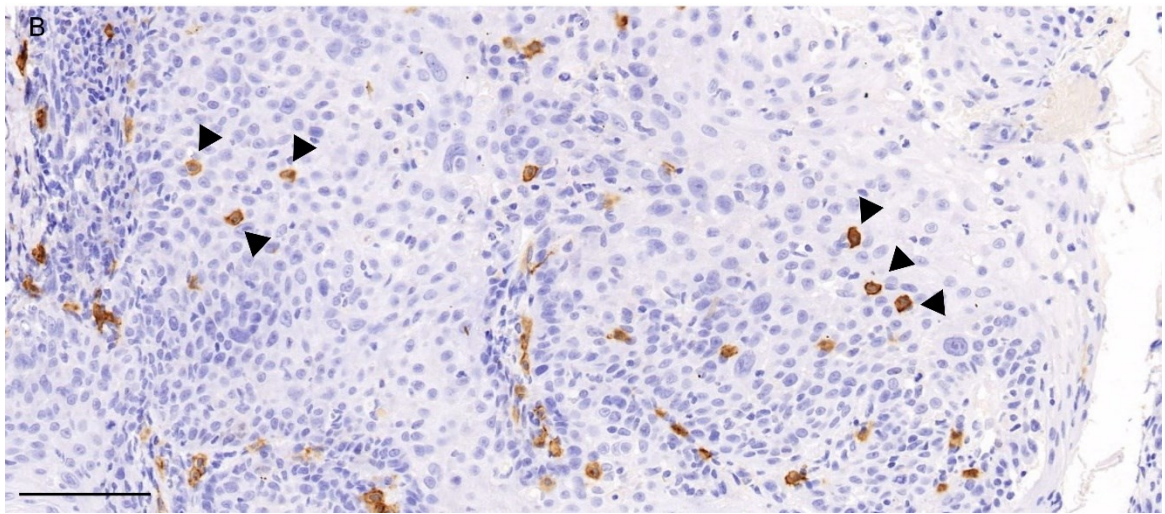

Supplement: Supplementary file 1 [file genes-16-01275-s001.zip › PVL immunohistochemical study- Figure S1.pdf]
